# The Lung Microbiome of Ugandan HIV-Infected Pneumonia Patients Is Compositionally and Functionally Distinct from That of San Franciscan Patients

**DOI:** 10.1371/journal.pone.0095726

**Published:** 2014-04-21

**Authors:** Shoko Iwai, Delphine Huang, Serena Fong, Leah G. Jarlsberg, William Worodria, Samuel Yoo, Adithya Cattamanchi, J. Lucian Davis, Sylvia Kaswabuli, Mark Segal, Laurence Huang, Susan V. Lynch

**Affiliations:** 1 Division of Gastroenterology, University of California San Francisco, San Francisco, California, United States of America; 2 School of Public Health, University of California Berkeley, Berkeley, California, United States of America; 3 HIV/AIDS Division, University of California San Francisco, San Francisco, California, United States of America; 4 Division of Pulmonary and Critical Care Medicine, University of California San Francisco, San Francisco, California, United States of America; 5 Department of Medicine, Mulago Hospital, Makerere University, Kampala, Uganda; 6 Department of Epidemiology and Biostatistics, University of California San Francisco, San Francisco, California, United States of America; University of California Los Angeles, United States of America

## Abstract

Sub-Saharan Africa represents 69% of the total number of individuals living with HIV infection worldwide and 72% of AIDS deaths globally. Pulmonary infection is a common and frequently fatal complication, though little is known regarding the lower airway microbiome composition of this population. Our objectives were to characterize the lower airway microbiome of Ugandan HIV-infected patients with pneumonia, to determine relationships with demographic, clinical, immunological, and microbiological variables and to compare the composition and predicted metagenome of these communities to a comparable cohort of patients in the US (San Francisco). Bronchoalveolar lavage samples from a cohort of 60 Ugandan HIV-infected patients with acute pneumonia were collected. Amplified 16S ribosomal RNA was profiled and aforementioned relationships examined. Ugandan airway microbiome composition and predicted metagenomic function were compared to US HIV-infected pneumonia patients. Among the most common bacterial pulmonary pathogens, *Pseudomonas aeruginosa* was most prevalent in the Ugandan cohort. Patients with a richer and more diverse airway microbiome exhibited lower bacterial burden, enrichment of members of the Lachnospiraceae and sulfur-reducing bacteria and reduced expression of TNF-alpha and matrix metalloproteinase-9. Compared to San Franciscan patients, Ugandan airway microbiome was significantly richer, and compositionally distinct with predicted metagenomes that encoded a multitude of distinct pathogenic pathways e.g secretion systems. Ugandan pneumonia-associated airway microbiome is compositionally and functionally distinct from those detected in comparable patients in developed countries, a feature which may contribute to adverse outcomes in this population.

## Introduction

Emerging evidence in the nascent, though rapidly evolving field of human microbiome research has consistently demonstrated the presence of polymicrobial communities on mucosal surfaces, including the respiratory tract [1]–[5]. Moving beyond description of these communities, a number of recent studies have demonstrated relationships between microbiome composition and features of pulmonary disease [3], [6], implicating the airway microbiome in respiratory disease pathogenesis. At other mucosal sites e.g. the gut, microbiome composition has been shown to define host susceptibility to infection [7], [8], a feature that has also been recently demonstrated in the upper respiratory tract, where the composition of the sinus mucosa microbiome influences pathogen abundance and their capacity to cause infection [9], indicating that the composition of the mucosal microbiome may define susceptibility to pathogen invasion and infection.

We have previously demonstrated that HIV-infected patients with acute pneumonia possess relatively diverse lower airway microbial communities comprised of multiple distinct taxa, including many known pathogens associated with HIV-infected populations [4]. However, even in the absence of acute respiratory infection, the lower airways of HIV-infected patients exhibit the presence of a relatively diverse bacterial community [10]. In contrast, healthy subjects exhibit little evidence of microbial presence at this site [3], [10], indicating that HIV-infection, which itself is a risk factor for developing pulmonary infection, is associated with lower airway colonization by microbial species.

Sub-Saharan Africa represents a large majority (69%) of the total number of individuals living with HIV infection worldwide and, in the most recent report, accounted for 72% of AIDS deaths globally [11]. Pulmonary infection represents a common and frequently fatal complication in this population, though little is known regarding the microbiome composition of the lower airways of these patients. Here, we examine the lower airway microbiome present in bronchoalveolar lavage (BAL) samples from a cohort of 60 HIV-infected Ugandan patients with acute pneumonia. Our objectives were to describe the communities of bacteria in sub-Saharan African patient population, and determine whether relationships exist between these assemblages and measured demographic, clinical, immunological and microbial variables. In addition, we sought to compare and contrast Ugandan and San Franciscan lower airway microbiome in an attempt to determine whether geography influences the composition of bacterial communities of HIV-infected patients in developing and developed nations.

## Methods

### Ethics Statement

The UCSF Committee on Human Research, the Makerere University School of Medicine Research Ethics Committee, the Mulago Hospital Research and Ethics Committee, and the Uganda National Council for Science and Technology approved the protocol. Subjects provided written, informed consent.

### Subjects

HIV-infected subjects (n = 60) were admitted to Mulago Hospital, Kampala, Uganda for acute pneumonia between October 2009 and October 2010 (Table 1). Each patient underwent two sputum acid fast bacilli (AFB) smear examinations to diagnose pulmonary TB. AFB smear-negative patients underwent bronchoscopy with BAL for clinical diagnosis.

**Table 1:** Clinical characteristics of subjects examined and differences between the Ugandan and San Franciscan patient populations.

Variable	San Francisco (N)	Uganda (N)	Between two sites p-value†
***Clinical variables***			
Age (Median, Min-Max)	43, 27–51 yrs (15)	33, 19–80 yrs (60)	0.018*
Gender	27% Female (15)	53% Female (60)	0.12
CD4^+^ cell count (Median, Min-Max)	36, 2–305 (15)	58, 1–454 (58)	0.26
Antiretroviral treatment (ART)	27% (15)	15% (60)	0.49
Days on ART (Median, Min-Max)	NA	0, 0–1183 (60)	NA
Smoke >100 cigarettes entire life	NA	27% (60)	NA
Ever had alcohol	NA	55% (60)	NA
Confirmed TB	0% (15)	58% (60)	0.00017***
Probable Bacterial Pneumonia or Bronchitis	47% (15)	18% (40)	0.062
Confirmed PCP	47% (15)	3% (60)	<0.0001***
Confirmed PKS	6.7% (15)	6.7% (60)	1
Confirmed fungal pneumonia	6.7% (15)	0% (60)	0.45
Confirmed cytomegalovirus pneumonitis	6.7% (15)	NA	NA
30day Survived after BAL	NA	82% (45)	NA
***Microbiological variables***			
Richness (Number of detected taxa on chip; Median, Min-Max)	417, 270–613 taxa (15)	742, 216–1273 taxa (60)	<0.0001***
Faith’s Phylogenetic Diversity (Median, Min-Max)	2.17, 1.58–3.06 (15)	3.62, 1.51–5.23 (60)	<0.0001***
Bacterial burden (16S rRNA copies/20 ng DNA; Median, Min-Max)	2.53×10^3^, 2.26×10^2^−8.06×10^4^ (15)	8.29×10^3^, 1.71×10^2^−3.62×10^5^ (60)	0.068

† p-values were calculated by either t-test (if equal variance), Welch’s test (if not equal variance) or proportion test as appropriate.

*p<0.05,

**p<0.01,

***p<0.001.

HIV-infected subjects (n = 15) enrolled in a previous study [4] were admitted to San Francisco General Hospital for acute pneumonia from July 2008 through October 2009. Patients underwent bronchoscopy with BAL for clinical diagnosis as described previously [4].

### Sample and Clinical Data Collection

Bronchoscopy with BAL was performed in the sub-segment that was most involved on chest imaging or the right middle lobe if the imaging revealed diffuse pneumonia. For Ugandan specimens, a fifteen ml aliquot of BAL fluid was promptly mixed with 30 mL of RNAlater solution (Life Technologies, Carlsbad, CA) and placed at 4°C for 16 hours, prior to storage at −80°C until processed for microbiome profiling. For San Franciscan specimens, a 3–5 ml aliquot of BAL was immediately placed on ice following collection and stored at −20°C until processed. Efforts to minimize the potential for oral microbiome contamination in airway samples included an oral decontamination step which includes scraping the patient’s tongue 6–8 times with a sterile tongue depressor and having them rinse with 10 ml of sterile 0.9% NaCl for 60 seconds followed by 0.12% chlorhexidine gluconate for 60 seconds immediately prior to bronchoscopy, as we have performed in a previous study in which we observed distinct microbiome in oral and airway samples [4]. This approach has been adopted as part of the current protocols of the National Heart Lung and Blood Institute’s multicenter Lung HIV Microbiome Project [12]. Clinical data were collected using standardized forms and included patient demographics, habits, prior or underlying lower airway disease, CD4^+^ cell count (considered either as a continuous or categorical variable; <200 or ≥200), antiretroviral therapy (ART), and antibiotic administration (Table S1 in File S2). As in prior studies, clinical diagnoses were assigned according to pre-specified criteria (described below) two months after discharge. Using these criteria, the following diagnoses were made in the Ugandan cohort under study: *Confirmed TB (n = 35):* Positive sputum or BAL culture on Lowenstein Jensen media or positive GeneXpert. *Confirmed PCP (n = 2):* Positive BAL microscopic examination using Diff-Quik. *Confirmed pulmonary Kaposi sarcoma (n = 4):* Characteristic Kaposi sarcoma lesions seen on bronchoscopic inspection of endobronchial tree. *Probable bacterial pneumonia or bronchitis (n = 7):* Clinical and radiographic presentation suggestive of bacterial pneumonia, response to empiric antibiotic therapy, and no alternate microbiologic diagnosis.

### DNA and RNA Extraction, 16S rRNA Gene Amplification and Profiling

Total DNA and RNA from BAL samples were extracted in parallel using the AllPrep DNA/RNA extraction kit (Qiagen, Hilden, Germany) as described previously [4]. Extracted RNA was stored in 80% ethanol at −80°C until used for analyses. The 16S rRNA gene was amplified using bacterial universal primers; 27F and 1492R [13] using twelve PCR reactions per sample run across a gradient of annealing temperatures (47°C–58°C) as described previously [4]. All PCRs were performed with parallel no-template control reactions in which no amplified product was observed. PCR product purification, fragmentation and hybridization to the G2 16S rRNA PhyloChip were performed as described previously [4]. The PhyCA algorithm [14] with customized *r score* cutoff values. Quartile *r score* cutoffs were chosen to rQ_1_>0.31, rQ_2_>0.56 and rQ_3_>0.80 based on fluorescence intensities of spiked-in control probes as described previously [15]. The PhyloChip data have been deposited and are publicly available (accession number: GSE52791).

### Quantification of 16S rRNA

16S rRNA gene copy number was assessed by quantitative PCR (Q-PCR) using the 16S rRNA universal primers and TaqMan probes; P891F (5′-TGGAGCATGTGGTT TAATTCGA-3′), P1033R (5′-TGCGGGACTTAACCCAACA-3′) and UniProbe (5′-FAM-CACGAGCTGACGACARCCATGCA-BHQ-3′; [16]). Total 16S rRNA gene copy number was calculated against a standard curve of known 16SrRNA copy numbers (1×10^2^−1×10^9^). Regression coefficients for all standard curves were >0.99. Q-PCR was performed in triplicate 20 µl reactions containing 1×TaqMan Universal Master Mix (Life Technologies), 20 ng of extracted DNA, each primer at a final concentration of 900 nM and UniProbe at a final concentration of 125 nM under the following conditions: 95°C for 10 min, followed by 40 cycles of denaturation at 95°C for 15 s and annealing and extension at 60°C for 1 min. No-template control reactions run in parallel did not produce any detectable signal.

### Gene Expression Analysis

RNA was treated with DNase using Turbo DNA-free (Life Technologies) according to the manufacturer’s protocol. The absence of DNA was verified by PCR with bacterial universal 16S rRNA primers (27F and 1492R; see above) as well as human 18S rRNA primers; 18S_4F (5′-CTGGTTGATCCTGCCAGTAG-3′) and 18S_1264R (5′-GAGGTTTCCCGTGTTGAGTC-3′). RNA quality was assessed using a Bioanalyzer and the RNA 6000 Nano Kit (Agilent Technologies, Santa Clara, CA). cDNA was synthesized using random hexamers and Super Script III First-Strand Synthesis System (Life Technologies) according to the manufacturer’s instructions. Q-PCR was performed as described above for TaqMan assay or in triplicate 25 µl reactions containing 1×QuantiTect SYBR Green PCR master mix (Qiagen), 20 ng of extracted DNA, and each primer at a final concentration of 300 nM under the following conditions: 95°C for 15 min, followed by 40 cycles of denaturation at 95°C for 30 s, annealing at 60°C for 30 s, and extension at 72°C for 30 s. Melt curve analysis was examined in all runs to confirm detection specificity. GAPDH expression was used to normalize gene expression across samples. All primer sets used for expression analyses are provided in Table S2 in File S2.

### Statistical Analyses

Statistical analyses were performed in the R environment (www.R-project.org). Faith’s phylogenetic diversity was calculated using the *Picante* package [17]. Permutational Multivariate Analysis of Variance Using Distance Matrices (*Adonis*), non-metric multidimensional scaling (NMDS) was performed using a Canberra distance matrix with the ecological community analysis R package *vegan* (version 2.0–4). Correlation coefficients were calculated using R package, *Hmisc*. The Significance Analysis of Microarrays (SAM) package was used to perform penalized regression analyses. False discovery rate was calculated using a package, *q-value*.

### PICRUSt Functional Metagenome Prediction

Community functional metagenome predictions were facilitated using the Phylogenetic Investigation of Communities by Reconstruction of Unobserved States (PICRUSt; http://picrust.github.io/picrust/; [18]). Representative sequences of taxa detected by the PhyloChip were retrieved from Greengenes database (http://greengenes.secondgenome.com/) and were used as an input for PICRUSt to predict biological functions. A heatmap was produced to visualize the presence-absence data of predicted KEGG orthologs (KOs).

## Results

### Lower Airway Microbiome Composition of Ugandan HIV-infected Pneumonia Patients

A total of 2,671 taxa belonging to 42 phyla were detected in at least one of the 60 Ugandan BAL samples examined; of these, only 33 taxa were common to all 60 subjects. Those shared taxa belonged to the Bifidobacteriaceae, Prevotellaceae, and Rikenellaceae amongst others (Table S3 in File S2). We next examined the cohort for detection of seven of the most common bacterial pulmonary pathogens detected in HIV-infected patients: *Pseudomonas aeruginosa*, *Haemophilus influenzae*, *Staphylococcus aureus, Chlamydophila pneumoniae, Mycoplasma pneumoniae, Streptococcus pneumoniae* and *Legionella pneumophila* were detected in 49, 10, 1, 0, 0, 0 and 0 of the subjects, respectively. Though the most common etiology of bacterial pneumonia in HIV-infected populations in westernized nations is *S. pneumoniae* [19], the taxon represented by this species was not detected in any of these antibiotic-treated Ugandan samples, which may be due to reduction of *Streptococcus* numbers below the sensitivity of the array. Instead, *P. aeruginosa* represented the most frequently detected pulmonary pathogen associated with pneumonia in this cohort. All detected taxa are summarized in Table S3 in File S2.

### Factors Related to Lower Airway Microbiome Composition

**Figure 1:**
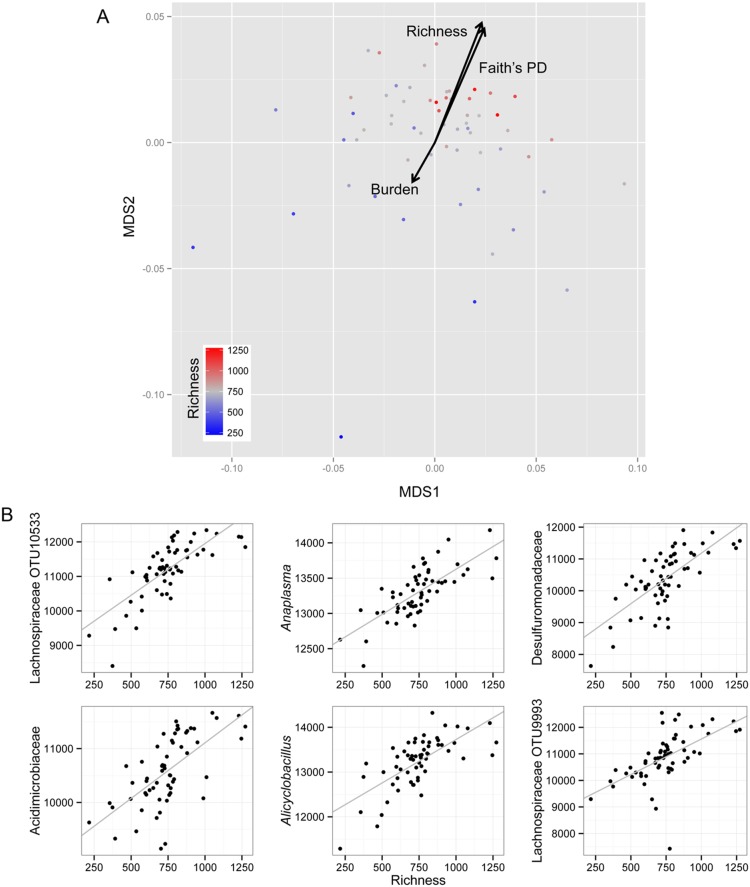
Variation in lower airway bacterial community composition is related to micobiological variables. **A.** NMDS ordination illustrating dis-similarity in bacterial community composition of 60 Ugandan HIV-infected patients with acute pneumonia. Degree of community richness (number of taxa detected in each sample) is indicated by color gradient. The ordination is overlaid with biplots to exhibit the observed relationships between bacterial community composition and bacterial richness, Faith’s phylogenetic diversity (PD) and bacterial burden. **B.** Top six taxa exhibiting the strongest significant positive correlations with microbial community richness (p<0.05; q<0.05).

To identify factors that explained the observed compositional variability in lower airway microbiome in HIV-infected Ugandan patients with acute pneumonia, we examined a variety of demographic, clinical and microbiological variables measured in our cohort (Table 2) by permutational analysis of variance using a distance matrix (*Adonis* in the vegan package). None of the demographic or clinical variables exhibited significant relationships with lower airway microbiome composition (Table 2). However, microbiological features including bacterial community richness (number of detected taxa in each sample), phylogenetic diversity (Faith’s index) and bacterial burden (total 16S rRNA copy numbers) were significantly associated with lower airway microbiome community composition (p = 0.001, 0.001, and 0.01, respectively; Table 2). NMDS ordination overlaid with bi-plots (Fig. 1A) was used to visualize these results, which indicated that bacterial burden was diametrically opposed to community richness and phylogenetic diversity. Spearman’s correlation confirmed that bacterial burden was negatively correlated with both community richness and phylogenetic diversity (r = −0.31, p<0.05, q<0.05 for both). Unsurprisingly, a strong positive correlation between community richness and phylogenetic diversity existed (r = 0.82, p<0.01, q<0.01). These data indicate that HIV-infected pneumonia patients with fewer types of organisms present tend to possess a much greater burden of bacteria in their lower airways.

**Table 2:** Relationships between measured clinical, immunological and microbiological variables and lower airway bacterial community composition in Ugandan HIV-infected patients.

Variable	N	Permutational analysis of variance (Adonis)		
		R2	p-value	q-value
***Clinical variables***				
Age	60	0.0052	0.99	0.69
Gender	60	0.018	0.34	0.49
CD4^+^ cell count (continuoous)	58	0.016	0.42	0.49
CD4^+^ cell count (<200 or >200)		0.015	0.50	0.49
Antiretroviral treatment (ART)	60	0.008	0.86	0.63
Days on ART	60	0.018	0.27	0.49
Smoke >100 cigarettes entire life	60	0.14	0.50	0.49
Ever had alcohol	60	0.030	0.099	0.24
Confirmed TB	60	0.013	0.57	0.49
Confirmed PCP	60	0.017	0.33	0.49
Confirmed PKS	60	0.013	0.53	0.49
Probable Bacterial Pneumonia or Bronchitis	40	0.014	0.83	0.63
30day Survived after BAL	45	0.012	0.86	0.63
***Microbiological variables***				
Richness (Number of detected taxa on the chip)	60	0.15	0.001***	0.0073
Faith’s phylogenetic diversity	60	0.16	0.001***	0.0073
Bacterial burden (16S rRNA copy numbers)	60	0.050	0.010	0.037
***Immunological variables***				
TNF-alpha	54	0.090	0.002**	0.0098
IL-6	54	0.017	0.48	0.49
Indoleamine 2,3-dioxygenase (IDO)	54	0.017	0.49	0.49
Matrix metalloproteinase (MMP)-9	54	0.048	0.027*	0.079
muc5AC	54	0.017	0.43	0.49

† *p<0.05,

**p<0.01,

***p<0.001.

To more specifically identify the bacterial species in species-rich and species-poor lower airway communities of HIV-infected pneumonia patients, we examined correlations between the reported relative abundance of each taxon detected and bacterial community richness. No taxa were significantly correlated with species-poor communities, perhaps due to outgrowth of distinct organisms under these conditions, hence the lack of significance for any one taxon. On the contrary, richer communities exhibited significant enrichment of members of the Lachnospiraceae, Desulfuromonadaceae, and Alicyclobacillaceae amongst others (Spearman’s correlation coefficient r = 0.68–0.78; p<0.0001, q<0.05; Figure 1B). All 1,952 taxa that were significantly correlated with community richness (p<0.05, q<0.05) are presented in Table S4 in File S2. These findings were reproduced by an independent univariate penalized regression approach, Significance Analysis of Microarrays (SAM), which also demonstrated that members of the Lachnospiraceae, Desulfovibrionaceae and Desulfuromonadaceae exhibited the strongest positive relationships with community richness (estimated p<0.05, q<0.05; Table S5 in File S2). These results indicate that presence of greater numbers of bacterial phylotypes and lower bacterial burden are characterized by significant enrichment of members of the Lachnospiraceae and a number of phylogenetically distinct sulfur-reducing bacteria, implicating these organisms in potentially controlling bacterial burden during antibiotic administration for acute respiratory infection in this cohort.

### Relationships between Lower Airway Microbiome and Host Markers of Inflammation

Based on this data, we hypothesized that compositionally distinct airway communities with lower bacterial burden are associated with a lower degree of airway inflammation and less mucosal damage. To address this hypothesis, we examined the expression of multiple host genes: inflammatory cytokines (IL-6, IL-17, TNF-alpha, IL-4 and IL-13), a mucin production gene (muc5AC), indoleamine 2,3-dioxytenase (IDO1; associated with impaired mucosal immunity and increased microbial translocation), and matrix metalloproteinase-9 (MMP9; abundant in lung diseases and involved in the breakdown of extracellular matrix). Gene expression data for each target locus were used as variables in permutational testing against community composition in *Adonis*. Amongst those genes examined, IL-17, IL-4 and IL-13 expression was not consistently detected across all 54 samples analyzed (only found in 81, 29 and 40% of samples respectively). When detected, expression was low, therefore these immunological variables were not considered in the subsequent permutational analysis. However, the expression level of TNF-alpha and MMP-9 exhibited significant relationships with bacterial community composition (p = 0.0020 and 0.027, respectively; Table 2).

**Figure 2:**
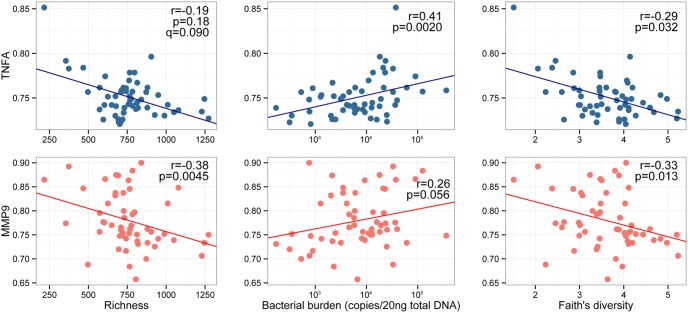
Gross features of lower airway microbiome are related to host TNF-alpha and MMP-9 expression. Spearman correlations indicate significant relationships between microbiological variables (community richness, Faith’s phylogenetic diversity and bacterial burden) and host gene expression (TNF-alpha and MMP-9). q<0.05 unless otherwise specified.

We next asked whether these two host gene expression profiles were related to other features of the microbiome. TNF-alpha expression was positively correlated with airway bacterial burden (r = 0.41, p<0.01, q<0.05) and negatively correlated with phylogenetic diversity (r = −0.29, p<0.05, q<0.05), though the relationship with community richness did not quite reach significance (r = −0.19, p = 0.18, q = 0.09; Figure 2). This indicates that TNF-alpha expression increases in parallel with increased bacterial burden and decreased phylogenetic diversity in the lower airways. To determine the specific taxa within the microbiome associated with this increase in TNF-alpha expression, we again calculated Spearman’s correlation coefficients. Following correction for false discovery, a member of the unclassified Acidobacteriaceae, Pasteurellaceae (represented by *Actinobacillus rossi*), and *Campylobacter* (represented by *Campylobacter insulaenigrae*) amongst others exhibited a significantly positive correlation with TNF-alpha expression (r = 0.34–0.47, p<0.05, q<0.05; Table S6 in File S2). On the other hand, unclassified Bradyhizobiaceae, Acidobacteriaceae, and Phyllobacteriaceae, amongst others were significantly negatively correlated with TNF-alpha expression (Spearman’s correlation coefficient r<−0.50, p<0.05, q<0.05; Table S6 in File S2).

MMP-9 expression, which is involved in airway remodeling, and was significantly negatively correlated with bacterial community richness and phylogenetic diversity (r = −0.38, p<0.005, q<0.05 and r = −0.33, p<0.05, q<0.05, respectively; Figure 2). Bacterial burden exhibited relatively weak positive correlation with MMP-9 expression, although this relationship did not quite reach significance (r = 0.26, p = 0.055, q<0.05; Figure 2). These results indicate increased expression of MMP-9 in association with species poor, less diverse and higher burden communities. Indeed, some of the strongest relationships between MMP-9 expression and taxon relative abundance were negative and included members of the genera *Burkholderia* (r = −0.44, p<0.001, q<0.05; Table S7 in File S2).

Collectively, these data indicate that Ugandan HIV-infected pneumonia patients that possess richer airway bacterial communities, enriched for members of the Lachnospiraceae, Desulfovibrionaceae, and Desulfuromonadaceae amongst other bacterial families, tend to possess lower bacterial burden, reduced TNF-alpha and MMP-9 expression. On the contrary, species poor communities depleted of phylogenetic diversity, exhibit much greater bacterial burden and are associated with increased TNF-alpha and MMP-9 expression and enrichment of Acidobacteriaceae, as well as Pasteurellaceae, and *Campylobacter* species, implicating these community members in the induction of pro-inflammatory and epithelial remodeling host responses under these conditions.

### The Lower Airway Microbiome of Ugandan and San Franciscan HIV-infected Pneumonia Patients

To gain insights into the commonalities and differentiating taxonomic features of lower airway microbiome of HIV-infected pneumonia patients in developing and developed nations, we compared and contrasted the data generated in this study (n = 60) with that generated from a cohort of previously examined San Franciscan HIV-infected pneumonia patients (n = 15) [4]. As San Franciscan samples collected in our previously published study were not stored in RNAlater, we first showed that this distinct sampling handling approach did not significantly influence community composition. An additional four BAL samples were collected from HIV-infected patients admitted to San Francisco General Hospital. Each sample was divided into two equal volumes. The first was centrifuged and the resulting cell pellet immediately placed at −80°C (P samples). The second aliquot was added to two volumes of RNALater, placed at 4°C overnight (to permit penetration of the sample by the preservative) prior to storage at −80°C (R samples). DNA was extracted from paired samples under identical conditions (as described in the materials and methods), and identically amplified 16S rRNA PCR products were analyzed by 16S rRNA PhyloChip as described in the materials and methods. Inter- and intra-sample Canberra distances between each pair of differentially processed samples and all other samples were examined. Inter-sample Canberra community distances between pairs of samples were significantly smaller than intra-sample distances (p<0.001), indicating that the sample collection method bias did not significantly impact community composition (Figure S1 in File S1).

**Figure 3:**
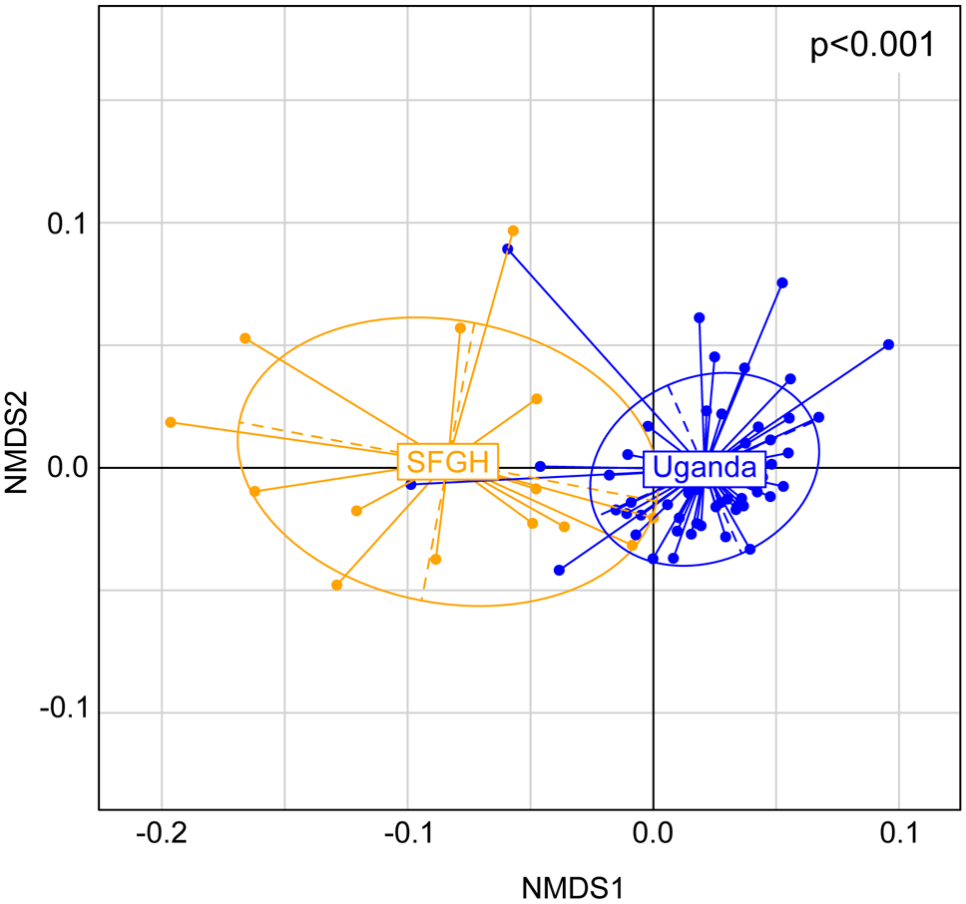
Ugandan and San Franciscan lower airway microbiome are compositionally distinct. NMDS ordination illustrates compositional dis-similarity in the lower airway microbiome of 60 Ugandan and 15 San Franciscan HIV-infected pneumonia patients.

The bacterial burden of Ugandan lower airway microbiome was comparable to that of San Franciscan participants, however, relative bacterial richness and phylogenetic diversity were significantly higher in Ugandan samples (Table 1; Figure S2 in File S1). Ordination of all 75 samples using NMDS based on a Canberra distance matrix exhibited significant separation of Ugandan and San Franciscan lower airway microbiome (*Adonis*; p<0.001; Figure 3). At the taxon-level, 1,203 taxa detected in Ugandan lower airway microbiome, were also found in at least one of the San Franciscan samples (Table S3 in File S2). Of these, only 20 taxa were common to all Ugandan and San Franciscan samples examined including members of Rikenellaceae, Bacillaceae and Lachnospiraceae amongst others. Those taxa appear to represent organisms consistently detected in the lower airways of HIV-infected pneumonia patients irrespective of the cause of their acute pulmonary infection. Comparative analyses indicated that 26 and 1,522 taxa were significantly (p<0.05, q<0.05) enriched in San Franciscan samples or Ugandan samples, respectively (Table S8 in File S2). San Franciscan patients’ microbiome were primarily enriched for members of the Firmicutes and Actinobacteria, in contrast, Ugandan patients exhibited significant expansion and enrichment of the Proteobacteria (p<0.05, q<0.05; Table S8 in File S2).

**Figure 4:**
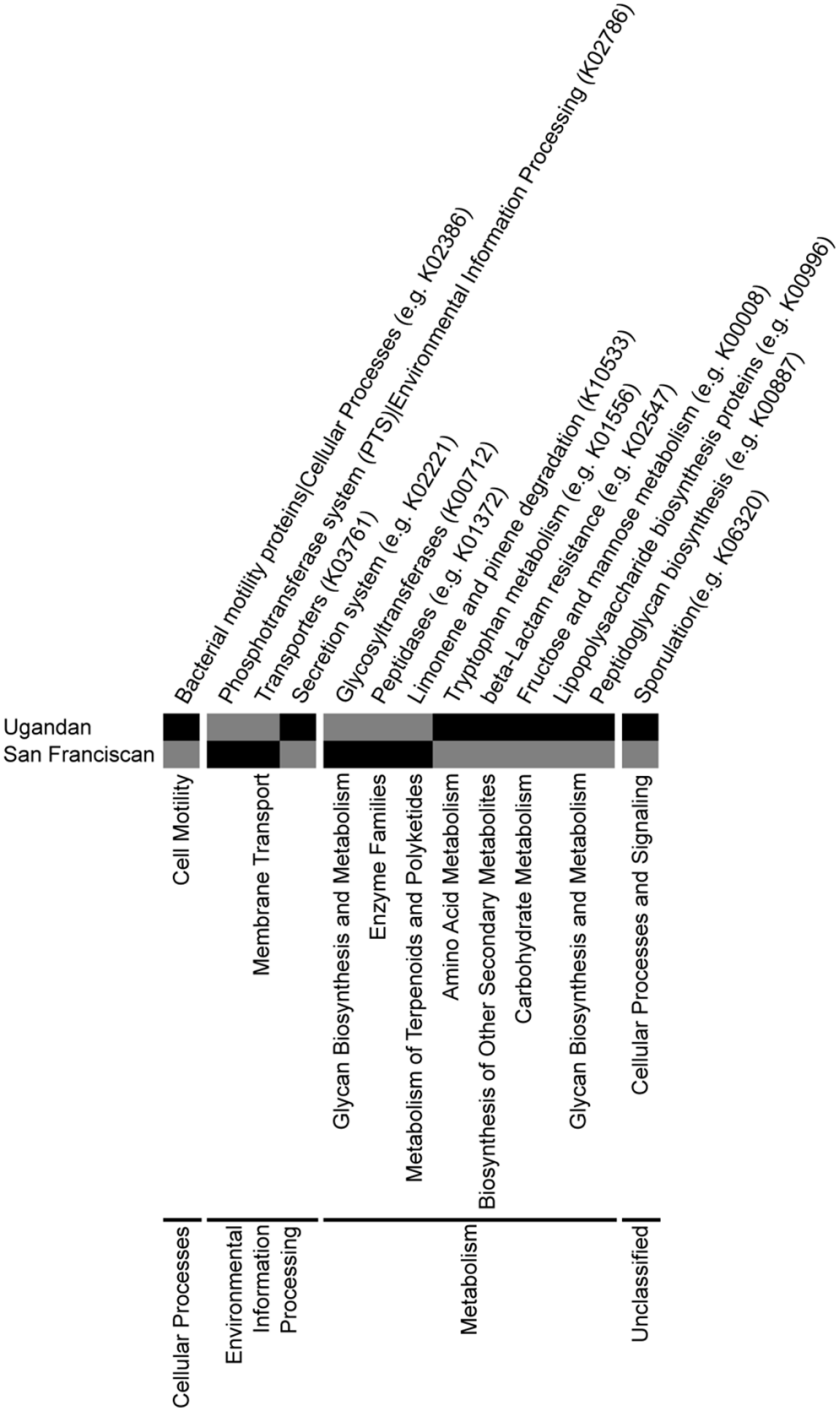
PICRUSt-based predicted metagenome indicates enrichment of multiple bacterial pathways involved in pathogenesis in Ugandan HIV-infected patients. Compared to San Franciscan HIV-infected patients, Ugandan HIV-infected patients possess lower airway microbial communities enriched in a variety of pathways involved in bacterial pathogenesis – a subset of the most commonly enriched pathways are shown. Black, present; gray, absent.

In an effort to cast some light on the functional implications of such distinct bacterial enrichments in Ugandan and San Franciscan HIV-infected pneumonia-associated lower airway microbiome, we predicted the functional gene pathways encoded by those taxa that were significantly enriched in either Ugandan or San Franciscan patients using reference 16S rRNA gene information. A total of 461 KEGG orthologs (KOs) were predicted to exist in both cohorts, which consisted primarily of pathways involved in fundamental microbial cell physiology and metabolic functions such as transporters and amino-acid metabolism (Table S9 in File S2). Only eight KOs were exclusive to San Franciscan samples including components of a lactose phosphotransferase system, glycan biosynthesis and polyketide degradation pathways (Table S10 in File S2). Unsurprisingly, given the number of taxa significantly enriched in Ugandan samples, 3,191 KOs were present exclusively in the Ugandan airway microbiome (Table S10 in File S2), highly enriched for KOs involved in microbial motility (flagellar assembly), bacterial secretion systems, tryptophan biosynthesis and metabolism, secondary metabolite biosynthesis, simple sugar metabolism, lipopolysaccharide and peptidoglycan biosynthesis and sporulation amongst others (Figure 4). The majority of the pathways enriched in this population are associated with pathogenic processes, suggesting that this sub-Saharan cohort of patients possess an airway microbiome that is functionally distinct from that present in western HIV-infected patients, and highly enriched in microbial pathogenesis pathways despite antimicrobial treatment for acute pneumonia.

## Discussion

We profiled lower airway microbiome in Ugandan HIV-infected, antibiotic-treated patients with acute pneumonia. To the best of our knowledge, this is the first detailed report of the lower airway microbiome of an African HIV-infected cohort. Limitations of the study include heterogeneity of the Ugandan HIV-infected population and limited information regarding long-term outcomes. Nonetheless, our results indicate the presence of polymicrobial communities in these acutely infected patients despite administration of a wide range of antimicrobial therapies. This phenomenon was also observed in a cohort of HIV-infected patients resident in San Francisco [4], as well as a cohort of non HIV-infected San Franciscan pneumonia patients [20]. This suggests that multiple species are capable of withstanding the effects of antimicrobial treatment, though it should be cautioned that the assays in these studies are DNA-based and not necessarily reflective of viable organisms in this niche.

In the Ugandan cohort, *Pseudomonas* was more frequently detected compared to other common respiratory pathogens. It should be noted that all but one patient (UG1690; n = 59) were administered at least one class of antimicrobial therapy within the two weeks prior to BAL collection. This suggests that antimicrobial administration may promote the enrichment of *P. aeruginosa* in the lower airways, a phenomenon which has been previously observed to occur and persist for months in longitudinally sampled non-HIV infected pneumonia populations [20]. This may also be due to the particularly low CD4^+^ cell counts (median 58, range 1–454) exhibited by these advanced stage HIV-infected patients, a factor previously associated with increased prevalence of *P. aeruginosa* pneumonia in this population [21], [22].

Although clinical variables did not explain the variability in lower airway microbiome composition, microbiological factors did reach significance. Patients with fewer detected taxa and lower phylogenetic diversity exhibited greater bacterial burden, moreover these communities were also associated with higher TNF-alpha and MMP-9 expression levels. On the contrary, relatively richer communities exhibited lower bacterial burden and reduced expression of these pro-inflammatory and tissue repair genes. Given this data, it is tempting to speculate that reduced bacterial competition in the lower airways may permit species outgrowth leading to pro-inflammatory responses and increased mucosal damage. Indeed, in our previous airway microbiome study of seven non HIV-infected pneumonia patients, we observed that the only patient that clinically improved in their acute respiratory infection was also the only individual to recover community diversity in their airways [20]. Certainly the bacterial community composition surrounding an infectious species influences its behavior. This has been shown in several recent studies in the gut [8], [23], [24] as well as study by Abreu and colleagues [9], who demonstrated that depletion of the upper respiratory microbiome of mice resulted in increased pathogenicity of a normally commensal *Corynebacterium* species.

The fact that there was no one taxon that characterized communities with low relative richness and high bacterial burden suggest that a number of distinct pathogenic species were enriched in these communities resulting in a lack of statistical significance. Indeed, our ordination plots indicate a large amount of variability across samples with reduced richness and bacterial burden (Figure 1A), supporting this hypothesis. Interestingly, organisms associated with relatively high community richness included a preponderance of Lachnospiraceae, Streptomycetaceae members and phylogenetically distinct sulfur reducing bacteria. Lachnospiraceae belong to the order Clostridiales, a group of organisms synonymous with immune modulation [24], specific members of which have recently been shown to induce anti-inflammatory T-regulatory cells [25], [26] In addition, hydrogen sulfide, known to be produced by sulfur-reducing bacteria, has been reported to be protective in lung injury models by preserving mitochondrial morphology and function in rat model [27]. Members of the Streptomycetaceae represent some of the most prolific producers of antimicrobial compounds (many of which are used clinically e.g. neomycin, chloramphenicol), supporting the hypothesis that these richer communities are naturally more competitive, and reduce the possibility of individual species proliferation.

The composition of lower airway microbiome of HIV-infected patients with pneumonia in Uganda was significantly different from those in San Francisco. These differences may be due to the observed differences in clinical status, age and/or pneumonia type across the geographically distinct cohorts (Table 1). Other emerging possibilities include diet, ethnicity (genetics) and environmental exposures in these developing and developed nations. A recent study demonstrated that environmental biodiversity is linked to skin microbiome composition and allergic disease development in western nations [28]. It is conceivable that environmental microbial exposures in distinct geographic regions play a role in defining microbiome composition, and hence, outcomes in these populations. This is particularly likely in the lower airways of chronically-infected HIV patients, especially following acute respiratory infection and antimicrobial exposure-a period in which the microbiome is undergoing the process of secondary succession and reassembling following antimicrobial perturbation.

Though predicted metagenomic analyses cannot provide information on the end product expression profile of these communities, it does provide insight into the core genomic capacity of the bacterial community present. It is remarkable that the predicted airway metagenome of the Ugandan cohort is so strikingly enriched for a broad array of pathways involved in pathogenesis, compared to that of San Franciscan patients and may provide insights as to why mortality rates are significantly higher in African HIV-infected populations [29]. Collectively, these data indicate that the relationship between immune status, patient outcomes and airway microbiome function requires substantially greater attention in future studies.

## Supporting Information

File S1 (DOCX)

File S2 (XLSX)
