# Supplementary material for: The Lung Microbiome of Ugandan HIV-Infected Pneumonia Patients Is Compositionally and Functionally Distinct from That of San Franciscan Patients
Source: PLoS One. 2014 Apr 21;9(4):e95726. doi: 10.1371/journal.pone.0095726 (PMC3994144; doi:10.1371/journal.pone.0095726)
Supplement: File S1 — (DOCX) [file pone.0095726.s001.docx]

**The Lung Microbiome of HIV-infected Pneumonia Patients in Sub-Saharan Africa**

Shoko Iwai, Delphine Huang, Serena Fong, Leah G. Jarlsberg, William Worodria, Samuel Yoo, Adithya Cattamanchi, J. Lucian Davis, Sylvia Kaswabuli, Mark Segal, Laurence Huang, and Susan V. Lynch on behalf of the Lung MicroCHIP Study

Supporting Information

**SUPPLEMENTARY FIGURE LEGENDS**

**Figure S1.** Hierarchical cluster analysis of paired P and R samples collected from four additional HIV-infected patients.

**Figure S2.** Community diversity metrics of San Franciscan and Ugandan lower airway microbiome; Bacterial burden (16S rRNA gene copies/20ng of total DNA); Faith’s phylogenetic diversity; Bacterial richness (number of taxa detected by 16S rRNA PhyloChip).

**Figure S1**

**Figure S2**
